# Characterization of the vaginal microbiota of dairy cows treated with probiotics

**DOI:** 10.1007/s11259-026-11616-3

**Published:** 2026-11-11

**Authors:** Aline Policarpo Baioco, Fabiane Pereira de Moraes, Sergio Farias Vargas Júnior, Arnaldo Diniz Vieira, Rafael Gianella Mondadori, Fábio Pereira Leivas Leite, Jeferson Vidart Ramos, José Mário Barichello, Ligia Margareth Cantarelli Pegoraro, Bernardo Garziera Gasperin, André Gustavo Cabrera Dalto, Monique Tomazele Rovani

**Affiliations:** 1 Programa de Pós-graduação em Ciências Veterinária (PPGCV), Universidade Federal do Rio Grande do Sul (UFRGS), Porto Alegre, RS Brasil; 2 Programa de Pós-graduação em Veterinária (PPGV), Universidade Federal de Pelotas (UFPel), Pelotas, RS Brasil; 3 Programa de Pós-graduação em Biotecnologia (PPGBiotec), Universidade Federal de Pelotas (UFPel), Pelotas, RS Brasil; 4 Programa de Pós-Graduação em Microbiologia e Parasitologia (PPGMPar), Universidade Federal de Pelotas (UFPel), Pelotas, RS Brasil; 5 Centro de Ciências Químicas, Farmacêuticas e de Alimentos (CCQFA), Universidade Federal de Pelotas, Pelotas, RS Brasil; 6 Embrapa Clima Temperado, Pelotas, RS Brasil

**Keywords:** Reproduction, Bovine, Lactic acid bacteria, Uterine health, Microbial ecology

## Abstract

Approximately 50% of dairy cows develop postpartum uterine diseases, significantly affecting reproductive performance and economic returns. Treatment is usually based on antibiotics; however, alternative approaches have been investigated. This study evaluated the effects of intravaginal supplementation with lactic acid bacteria (LAB) on the vaginal microenvironment, focusing on microbial viability, pH, and taxonomic composition. A total of 21 non-pregnant, multiparous and non-lactating Jersey cows with synchronized estrous cycle were used. Animals were randomly assigned to two groups: Probiotic group (PRO; *n* = 11) received two intravaginal capsules containing *Lactobacillus rhamnosus* and *Lactobacillus reuteri* at 4.5 × 10¹⁰ CFU/capsule; and Control group (CTR; *n* = 10), receiving two intravaginal capsules without LAB. The recovery of *Lactobacillus rhamnosus* was significantly higher in the PRO group (*p* < 0.0001) vs. CTR. No significant differences were observed between CTR and PRO groups in vaginal pH (*p* > 0.05). Microbiota analysis based on the 16 S rRNA gene showed no significant differences in differential abundance between CTR and PRO groups at the genus level (q > 0.05). However, PERMANOVA with paired design revealed a significant effect of the group x timepoint model on microbial community composition (*p* = 0.004). This is the first study characterizing the vaginal microbiota of cows treated with probiotic LAB strains. *Lactobacillus rhamnosus* and *Lactobacillus reuteri* at the tested concentration did not alter vaginal pH up to Day 14 and did not significantly change individual taxon abundances at Days 4 or 7; however, paired analysis detected a significant compositional shift associated with treatment.

**Supplementary Information:**

The online version contains supplementary material available at https://doi.org/10.1007/s11259-026-11616-3.

## Introduction

The dairy industry plays a crucial role in global nutrition, not only due to the nutritional value of its products but also because it supports the livelihoods of millions and represents a major economic sector in many countries (FAO 2013; Fiorillo and Amico 2024). Within this context, ensuring animal health is fundamental to maintaining productivity and sustainability in dairy production systems. Traditionally, veterinary medicine has focused on disease diagnosis and therapeutic treatment with antibiotics. However, this approach can result in drug residues in animal-derived food products and contribute to the development of cross-resistance between zoonotic pathogens and human-associated microorganisms (ECDC et al. 2024). To address these concerns, a broader concept of health management has emerged- one that emphasizes disease prevention, productivity enhancement, and health promotion. This integrated approach also encompasses animal welfare, public health, and environmental sustainability (Gunnarsson 2006).

Among the key factors influencing dairy herd productivity, reproductive health stands out as a critical component (Inchaisri et al. 2010). Reproductive performance is closely tied to overall health, particularly during the transition period surrounding calving. In this regard, reproductive disorders, especially postpartum uterine diseases, have significant implications for both animal welfare and economic outcomes. It is estimated that approximately half of all dairy cattle experience uterine disease after calving, negatively affecting fertility, milk yield, and longevity in the herd (Sheldon et al. 2009). The associated economic losses are substantial; a recent study indicated that, when all reproductive disorders were considered, the mean annual cost of €20,013 per herd or €100 per cow. At the herd level, the highest mean annual cost was observed for acute metritis (€30/cow per year). This economic impact was mostly attributable to milk production losses, culling, veterinary services, and treatments (Wicaksono et al. 2025). Therefore, improvements in the prophylaxis and early diagnosis of uterine diseases are needed.

The established uterine microbiota changes rapidly postpartum, favoring anaerobic bacteria such as *Bacteroidetes* and *Fusobacteria* (Jeon et al. 2015). Shifts in the microbiome are a key factor in the development and resolution of uterine diseases. It is believed that the vaginal microbiota is directly associated with the development of uterine diseases such as metritis and endometritis (Jeon et al. 2015; Bicalho et al. 2017). Previous evidence indicates that strains of *Lactobacillus* suppress the growth of other endogenous bacteria in the human vagina through the production of organic acids such as lactic acid, hydrogen peroxide, and bacteriocins (Aroutcheva et al. 2001). Although data on cattle are limited, similar mechanisms are hypothesized to occur in cows.

In cattle, *Lactobacillus* species are present in the vaginal microbiota, although typically in low abundance. Recent research indicates that modulation of the vaginal microbiome may represent an alternative to antibiotic therapies. Probiotics are defined as ‘live microorganisms that, when administered in adequate amounts, confer a health benefit on the host’ (FAO/WHO 2001; Hill et al. 2014). The use of intravaginal probiotics may play an important role in the preventive management of postpartum uterine diseases (Adnane and Chapwanya 2022). The benefits of intravaginal probiotics have been evaluated in relation to the incidence of postpartum diseases, milk production, and the expression of inflammatory markers (Ametaj et al. 2014; Deng et al. 2015, 2016; Genís et al. 2017, 2018; Madureira et al. 2023). The strains used in the present study (*L. rhamnosus* LR04 and LR06 and *L. reuteri* LRE02) are commercially available probiotic strains; LR04 and LRE02 have documented probiotic use and have previously been used in combination (Drago et al. 2020), and LR04 has reported activity against *Escherichia coli* (Mogna et al. 2012; Genís et al. 2018), while related LAB combinations containing *L. rhamnosus* and *L. reuteri* have been administered intravaginally in dairy cows (Genís et al. 2017, 2018). However, unlike the human vaginal environment, which is characterized by a low pH, the bovine vagina maintains a near-neutral pH. Therefore, the rationale for using these strains rely not primarily on acidification, but on their potential for competitive exclusion, adhesion to the epithelium, and production of antimicrobial substances to inhibit pathogens.

The benefits of intravaginal probiotics have been evaluated in relation to the incidence of postpartum diseases, milk production, and the expression of inflammatory markers (Ametaj 2014; Deng et al. 2015, 2016; Madureira 2023). However, the viability of probiotics and their effects on pH and the microbiota in treated cows via this route of administration have not been described. Therefore, the aim of this study was to evaluate the effects of intravaginal probiotics based on a combination of *Lactobacillus* (*L. rhamnosus* and *L. reuteri*) on the vaginal microenvironment, with a focus on microbial viability, pH, and taxonomic composition.

## Materials and methods

### Animals

The study was carried out at Embrapa Clima Temperado, located in southern Brazil (31°49’02.1"S 52°27’14.2"W). Twenty-one non-pregnant Jersey multiparous cows, raised in an extensive pasture system, and non-lactating, with a mean body condition score (BCS) of 3.5 (on a scale of 1 to 5), were used in this study. The animals underwent a complete gynecological examination, including ultrasonographic evaluation to assess the uterus and ovaries and determine the reproductive status; only healthy animals were used in the study. All animals were handled according to the guidelines established by the Ethics and Animal Experimentation Committee, and the experimental protocol was approved by the Universidade Federal do Rio Grande do Sul (Protocol No. 44088).

### Experimental design

**Fig. 1:**
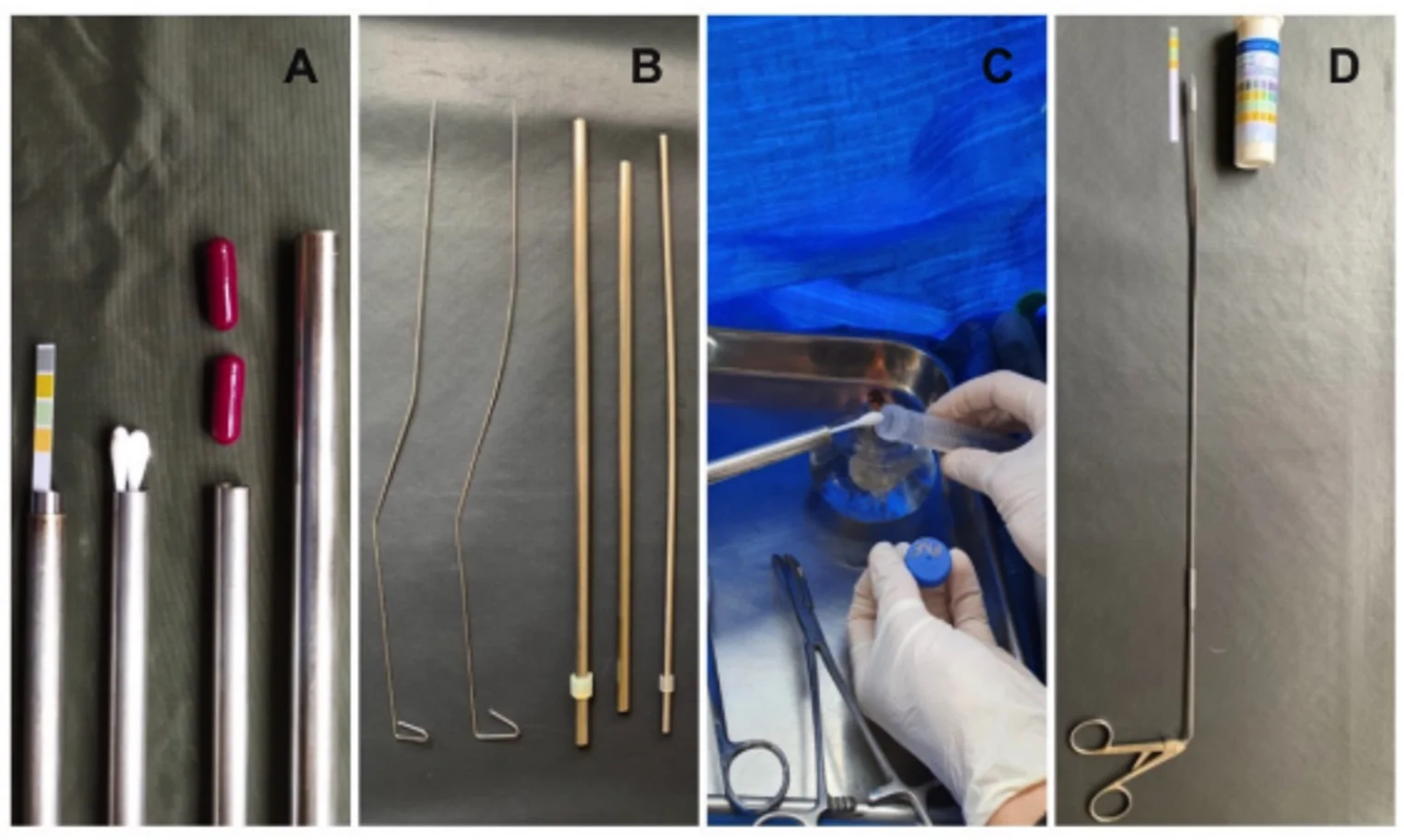
Capsules used for treatment administration (**A**). Intravaginal device used for swab collection (**B**). Post-collection swab stored in a Falcon tube containing MRS medium for microbiological culture (**C**). Hartmman forceps used for insertion of pH strip into the vagina (**D**)

All animals received an intramuscular (IM) injection of 150 mg of long-acting progesterone (Sincrogest^®^, Ouro Fino, Cravinhos, São Paulo, Brazil) 10 days before the start of treatments (D-10), to ensure that all animals were in similar reproductive endocrine conditions, since hormones can alter vaginal microbial composition (Bo et al. 1995). Progesterone injections were administered weekly until D14, totaling four administrations during the experimental period. Cows were randomly allocated into two groups for intravaginal treatment on Day 0 (D0): Control (CTR; *n* = 10), in which the animals received only two gelatin capsules without lactic acid bacteria (LAB); and Probiotic (PRO; *n* = 11), in which the animals received two capsules containing the probiotic LAB strains. After cleaning the vulva, the capsules were aseptically deposited in the vaginal fornix using a device that allows sterilization between procedures (Fig. 1A).

### Probiotic strains

The probiotic treatment consisted of a mixture of lactic acid bacteria (LAB) including *Lactobacillus rhamnosus* LR06 (DSM 21981), *Lactobacillus rhamnosus* LR04 (DSM 16605) and *Lactobacillus reuteri* LRE02 (DSM 23878), isolated from humans, with a final cell count of 4.5 × 10¹⁰ CFU/capsule and a ratio among the three probiotics strains of 12:12:1, respectively. A mixture of lyophilized strains of *L. rhamnosus* and *L. reuteri* was used in this study based on previous literature (Genís et al. 2017, 2018; Madureira et al. 2023).

### Collection of vaginal swabs

Vaginal swabs were collected at capsule insertion (day 0; D0), D2, D3, D4, D7, and D14, using a specific device (Fig. 1B). After cleaning the vulva, the device was inserted into the vagina, and the outer tube was retracted to expose the swab. Vaginal samples were collected by rotating the swab in contact with the vaginal wall. The outer tube was then reinserted to cover the swab and avoid contamination at removal. The device was cleaned with 70% alcohol and sterilized with flame between animals. At each sampling timepoint (D0, D2, D3, D4, D7, and D14), each animal had five swabs collected: two swabs were placed in a Falcon tube containing MRS semi-solid agar (De Man, Rogosa and Sharpe medium) for transport, refrigerated (8 °C) for later microbiological culture (Fig. 1C); three other swabs were stored frozen (-20 °C) in 300 µl of molecular stabilizing solution (SEMol) for further microbiota analysis.

### Culture and identification

The collected swabs were inoculated onto Petri dishes containing MRS agar (De Man, Rogosa and Sharpe) and incubated under anaerobic conditions at 37 °C for 48 h. Colonies observed at 48 h were submitted to Gram staining. For this, approximately 40 µl of distilled water was added to the microscopy slide and a small part of the colony was transferred to distilled water with the aid of a bacteriological loop. The sample was homogenized, and fixation was performed using heat in a laminar flow hood.

The staining process was performed with crystal violet, followed by Lugol and acetone. Subsequently, after counterstaining with fuchsin, the slides were washed with distilled water and air-dried. Slides were analyzed by oil immersion microscopy at 1000x magnification. Morphology (cocci or bacilli) and coloration (purple/violet for Gram-positive and pink/red for Gram-negative) were evaluated. All samples positive for bacilli on Gram staining collected on D2 and D4 were sent for species identification by matrix-assisted laser desorption/ionization time-of-flight (MALDI TOF) in a commercial laboratory.

### 16S rRNA sequencing

Vaginal swab samples collected from cows in the CTR (*n* = 5) and PRO (*n* = 5) groups on D4 and D7 (as growth data indicated probiotic viability up to D4 and clearance by D7) were analyzed using a standard commercial kit for the specific matrix to extract the DNA (Nextera DNA Library Preparation Kit, Illumina), following the manufacturer’s protocol. Quality and quantification were evaluated by NanoDrop spectrophotometry (Thermo Scientific). A segment of approximately 460 bp of the hypervariable V3-V4 region of the 16 S rRNA gene was amplified using the primers 341 F/805R for bacterial analysis, with the following PCR conditions: initial denaturation at 95 °C for 3 min; followed by 25 cycles of denaturation at 95 °C for 30 s, annealing at 55 °C for 30 s and extension at 72 °C for 30 s, with a final extension at 72 °C for 5 min.

The amplicons were indexed using Illumina Nextera dual-index barcodes under the following conditions: initial denaturation 95 °C for 3 min; followed by 8 cycles at 95 °C for 30 s, annealing at 55 °C for 30 s and extension at 72 °C for 30 s, with a final extension at 72 °C for 5 min. The products were then purified, pooled, and subsequently sequenced on an Illumina NextSeq platform (Degnan and Ochman 2012), generating paired-end reads of 300 bp.

Bioinformatics pre-processing: Reads were subjected to quality control using fastp v0.23 (Chen et al. 2018), including Illumina adapter removal, primer trimming at the 5′ ends (fixed trimming of 17 bp in R1 and 21 bp in R2), quality filtering (Phred score ≥ 20 using a 4-bp sliding window), and removal of reads shorter than 150 bp. Filtered reads were then aligned against the *Bos taurus* reference genome (ARS-UCD1.3, Ensembl release 112) using Bowtie2 v2.5 (Langmead and Salzberg 2012) in the “very-sensitive” mode. Read pairs in which both mates failed to align to the bovine genome were retained as microbial reads (samtools v1.x, flags -f 12 -F 256). The average host alignment rate was 45.6% for D4 samples and 64.1% for D7 samples, highlighting the importance of host DNA removal in vaginal mucosal samples. A high host-to-microbe DNA ratio is a recognized challenge in bovine vaginal samples, where host-derived DNA reduces the effective sequencing depth available for microbial content (Ong et al. 2022). The difference in host alignment rates between D4 and D7 therefore translated into unequal effective microbial depth between timepoints, and these rates are reported explicitly so that this source of variation can be taken into account when interpreting the diversity estimates presented below.

Library preparation and DADA2 Processing: Microbial reads were processed using the DADA2 v1.30 pipeline (Callahan et al. 2016) in the R v4.x environment. Following visual inspection of quality profiles, reads were truncated to 270 bp (R1) and 200 bp (R2), with a maximum expected error threshold of 2 for both forward and reverse reads (maxEE = c (2, 2) and removal of reads containing ambiguous bases (maxN = 0). Error rates were estimated using the DADA2 parametric error model, followed by dereplication and inference of amplicon sequence variants (ASVs). For the 341 F/805R amplicon (464 bp including primers; Klindworth et al. 2013), the primer-trimmed insert is approximately 426 bp, so the truncation lengths applied here (270 bp for R1 and 200 bp for R2) give an expected forward-reverse overlap of approximately 44 bp. This exceeds the default minimum overlap of 12 bp required by the DADA2 mergePairs function (Callahan et al. 2016) and is therefore compatible with reliable merging of read pairs. Forward and reverse reads were merged, and chimeric sequences were removed using the consensus method (removeBimeraDenovo). Taxonomic assignment was performed using the DADA2 naïve Bayesian classifier with a minimum bootstrap confidence threshold of 80% against the SILVA v138.1 database (Quast et al. 2012). ASVs classified as chloroplasts, mitochondria, or lacking phylum-level assignment were removed from the final dataset.

### Vaginal pH measurement

Vaginal pH was measured using commercial strips (Kasvi) with a scale of 0 to 14, with intervals of 1. Each animal had its pH measured once a day, at D0, D2, D3, D4, D7, and D14, using a single pH test strip. The pH strip was attached to the tip of Hartmann forceps (Fig. 1D), which was inserted through the rigid tube previously used to collect the swabs, positioned at the entrance of the cervix and pressed against the lateral vaginal wall. The strips were kept in the vagina for approximately 10 s before removal and subsequent reading using the color chart provided by the manufacturer.

### Statistical analysis

The distribution of continuous data was analyzed using the Shapiro-Wilk test, and data were normalized when necessary. The pH data were analyzed by analysis of variance (ANOVA). The analysis was performed using the JMP statistical software (JMP Statistical Discovery LLC).

All ecological analyses were performed in R using the phyloseq v1.46 (McMurdie and Holmes 2013), vegan v2.6 (Oksanen 2022), and microbiome (Lahti and Shetty 2018) packages.

The genus *Ureaplasma* exhibited variable dominance and substantial inter-individual variability (SD = 38.9% in the PRO group at D4), all analyses were performed in parallel using both the complete dataset (Full Dataset) and a dataset without genus *Ureaplasma* (without *Ureaplasma*). This approach allowed us to evaluate the influence of this dominant taxon on the observed microbial community patterns.

Alpha diversity was evaluated using the Observed ASVs, Chao1, Shannon, and Simpson indices. The following statistical approaches were employed: (i) the Wilcoxon rank-sum test (Mann–Whitney U test; Mann and Whitney 1947) to compare groups at each time point; (ii) the Wilcoxon rank-sum test applied to the paired difference (ΔD7–D4) between groups, thereby accounting for the repeated-measures design; and (iii) the paired Wilcoxon signed-rank test to compare D4 and D7 within each group. All P-values were adjusted for multiple comparisons using the Benjamini–Hochberg procedure.

Beta diversity was evaluated by Principal Coordinates Analysis (PCoA) based on Bray–Curtis dissimilarity calculated from relative abundances and by Principal Component Analysis (PCA) following Centered Log-Ratio (CLR) transformation, which is appropriate for compositional data (Gloor et al. 2017). Differences in microbial community composition were assessed using PERMANOVA (adonis2, 999 permutations) with a group × timepoint model, performed both without restriction and with permutations constrained by animal (strata = animal_id) to account for the paired repeated-measures design. Analyses were conducted using both Bray–Curtis and Aitchison (CLR-transformed data with Euclidean distance) dissimilarity metrics. The homogeneity of multivariate dispersions was evaluated using BETADISPER.

Differential abundance was assessed using two complementary methods that account for the compositional nature of amplicon sequencing data. ANCOM-BC2 (Lin and Peddada 2020) was performed using a minimum prevalence threshold of 10%, a library size cutoff of 1,000 reads, structural zero detection, and Benjamini–Hochberg correction (q < 0.05). ALDEx2 (Fernandes et al. 2014), which models compositional uncertainty through Dirichlet sampling (128 Monte Carlo instances), was performed using the Wilcoxon test with Benjamini–Hochberg correction. Both analyses were conducted separately for each time point at the genus level. These methods were combined because they rely on different assumptions to handle compositional data: ANCOM-BC2 estimates and corrects for differences in sampling fraction across samples (Lin and Peddada 2020), whereas ALDEx2 models compositional uncertainty through centered log-ratio transformation and Dirichlet resampling (Fernandes et al. 2014). In a benchmark of differential abundance methods across 38 datasets, ALDEx2 and ANCOM-based approaches were among the most conservative and produced the most consistent results (Nearing et al. 2022); applying them in parallel therefore provides a stringent assessment in a dataset of this size.

Taxonomic composition was visualized using stacked bar plots at the phylum and genus levels, presented both for individual samples and as mean relative abundances by group and timepoint. All figures were generated using ggplot2 v3.5 and patchwork.

All comparisons were evaluated considering a significance level of 5% (*P* < 0.05).

## Results

### Culture

**Fig. 2:**
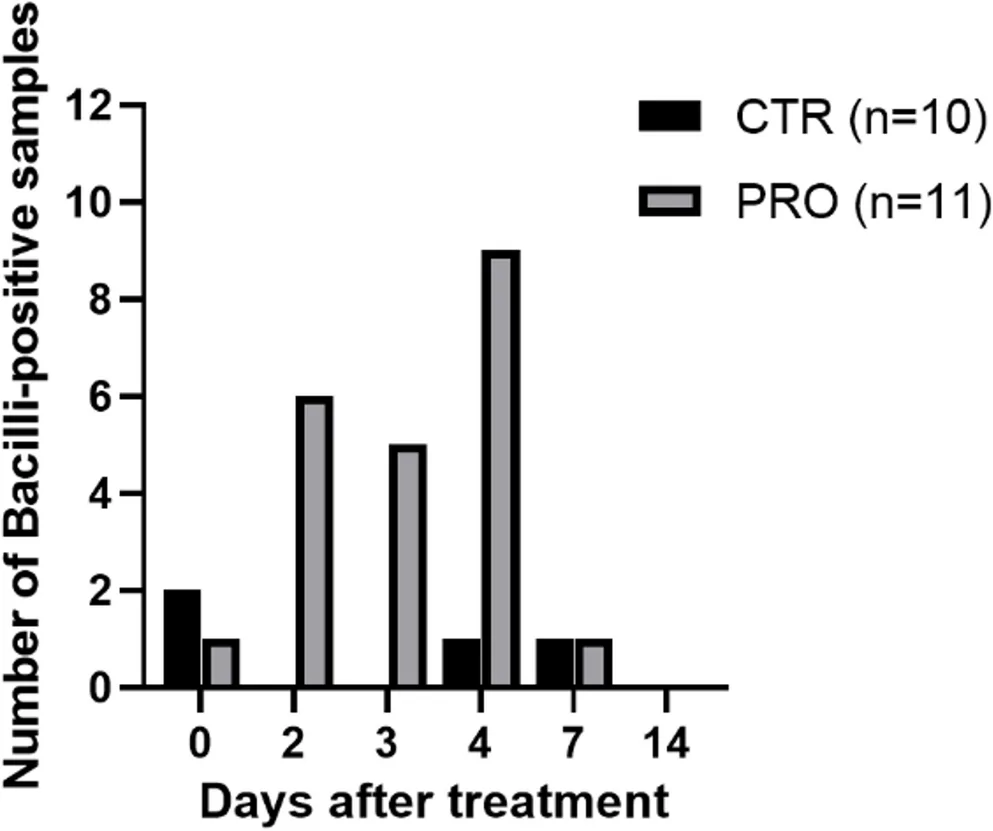
Number of Bacilli-positive samples in MRS medium throughout the experimental period in the Control (CTR; *n* = 10) and Probiotic (PRO; *n* = 11) groups on different days after treatment administration

Bacillary growth in culture was significantly higher in cows in the PRO group than in those in the CTR group on D2, D3 and D4 (Fig. 2; *P* < 0.0001), this difference was no longer detected on D7. The samples that showed growth in culture and were identified by Gram stain as bacilli were subsequently analyzed by MALDI-TOF (Table 1). However, only *Lactobacillus rhamnosus* was identified by MALDI-TOF; the *Lactobacillus reuteri* strain used in the capsule formulation was not detected. These results indicate that, of the two species administered, only *L. rhamnosus* was recovered by culture-based methods.

**Table 1:** Results of gram staining and species identification by matrix-assisted laser desorption/ionization time-of-flight (MALDI-TOF) on Day 2 and Day 4 after treatment administration in the Control (CTR; *n* = 10) and Probiotic (PRO; *n* = 11) groups

GROUP		Day 2		Day 4	
	COW	Gram	Maldi-TOF	Gram	Maldi-TOF
CTR	332	AG		bacillus +	*Bacillus cereus*
PRO	245	bacillus +	*Lactobacillus rhamnosus*	bacillus +	*Lactobacillus rhamnosus*
	381	AG		bacillus +	*Lactobacillus rhamnosus*
	502	bacillus +	*Lactobacillus rhamnosus*	bacillus +	*Lactobacillus rhamnosus*
	516	bacillus +	*Lactobacillus rhamnosus*	bacillus +	*Lactobacillus rhamnosus*
	522	bacillus	*Lactobacillus rhamnosus*	bacillus +	*Lactobacillus rhamnosus*
	537	bacillus +	*Lactobacillus rhamnosus*	bacillus +	*Lactobacillus rhamnosus*
	541	AG		bacillus +	*Lactobacillus rhamnosus*
	570	bacillus +	*Lactobacillus rhamnosus*	bacillus +	*Lactobacillus rhamnosus*
	602	AG		bacillus +	*Lactobacillus rhamnosus*

AG indicates absent growth. Cows without bacilli-positive cultures are not shown in this table

### Vaginal pH

**Fig. 3:**
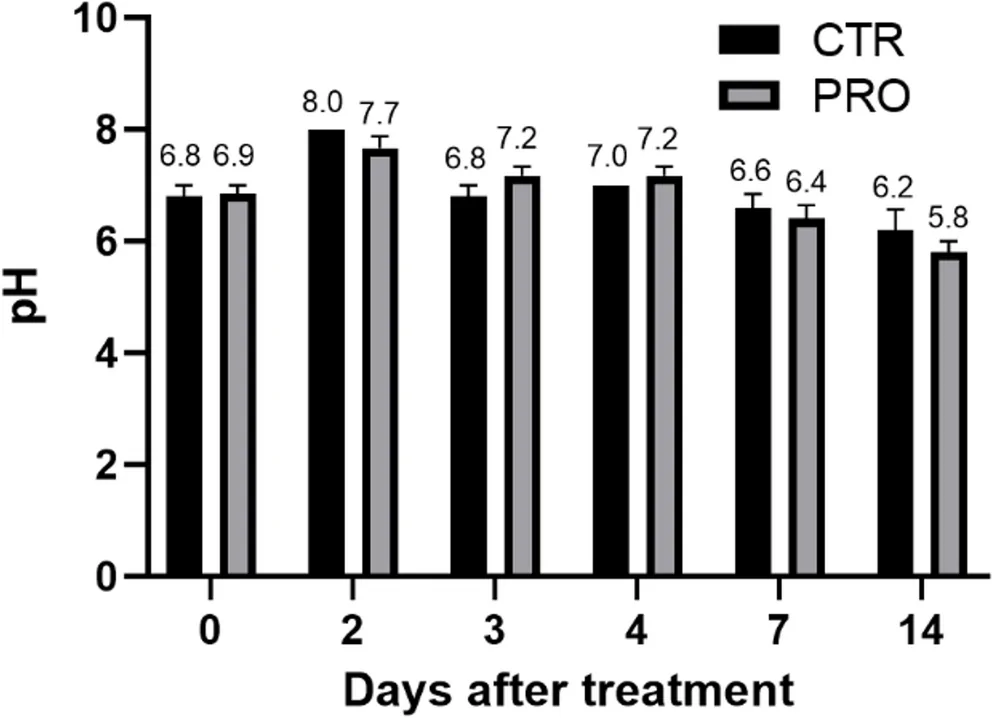
Vaginal pH in the Control (CTR; *n* = 10) and Probiotic (PRO; *n* = 11) groups on different days after treatment administration

The mean pH value of the CTR and PRO groups was 6.8 ± 0.2 and 6.9 ± 0.1, respectively. No significant differences in pH were observed between the groups at D0, D2, D3, D4, D7, and D14 (Fig. 3; *P* > 0.05).

### Microbiota study

#### Sequencing yield and quality control

Following quality control with fastp and removal of bovine host-derived reads, the DADA2 pipeline processed a total of 3,524,715 microbial read pairs from the 20 samples. After quality filtering (filterAndTrim), denoising, and chimera removal, 2,310,120 reads were retained, representing 9,711 ASVs assigned to 40 phyla and 666 genera. These totals include a large number of rare, low-prevalence ASVs; for this reason, differential abundance testing was restricted to taxa present in at least 10% of samples, as described in the Statistical Analysis section. Sequencing depth per sample ranged from 10,193 to 251,768 reads (Online Resource 1). Samples collected at D4 exhibited a higher mean sequencing depth than (188,730 reads/sample) those collected at D7 (49,854 reads/sample). Rarefaction curves indicated that most samples reached saturation for diversity sampling (Online Resource 2a). The dataset excluding *Ureaplasma* retained 9,645 ASVs and 1,789,592 reads across 665 genera, with sequencing depths ranging from 1,725 to 206,718 reads per sample (Online Resource 2b).

#### Alpha-diversity

**Fig. 4:**
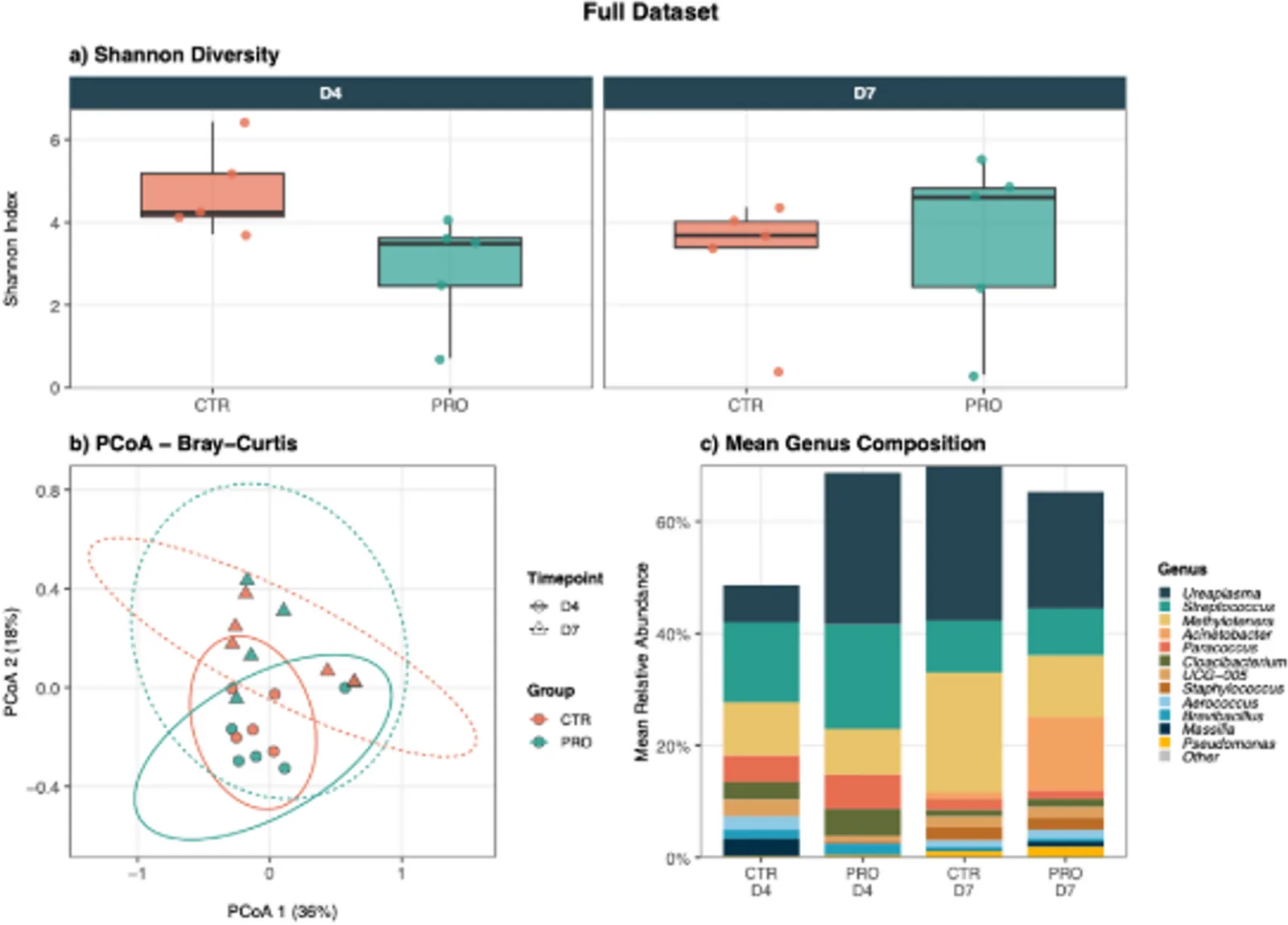
Shannon Diversity (**a**), PCoA – Bray- Curtis (**b**) and Mean Genus Composition (**c**) with Full Dataset from samples collected on Day 4 and Day 7 after treatment administration in the Control (CTR; *n* = 5) and Probiotic (PRO; *n* = 5) groups

**Fig. 5:**
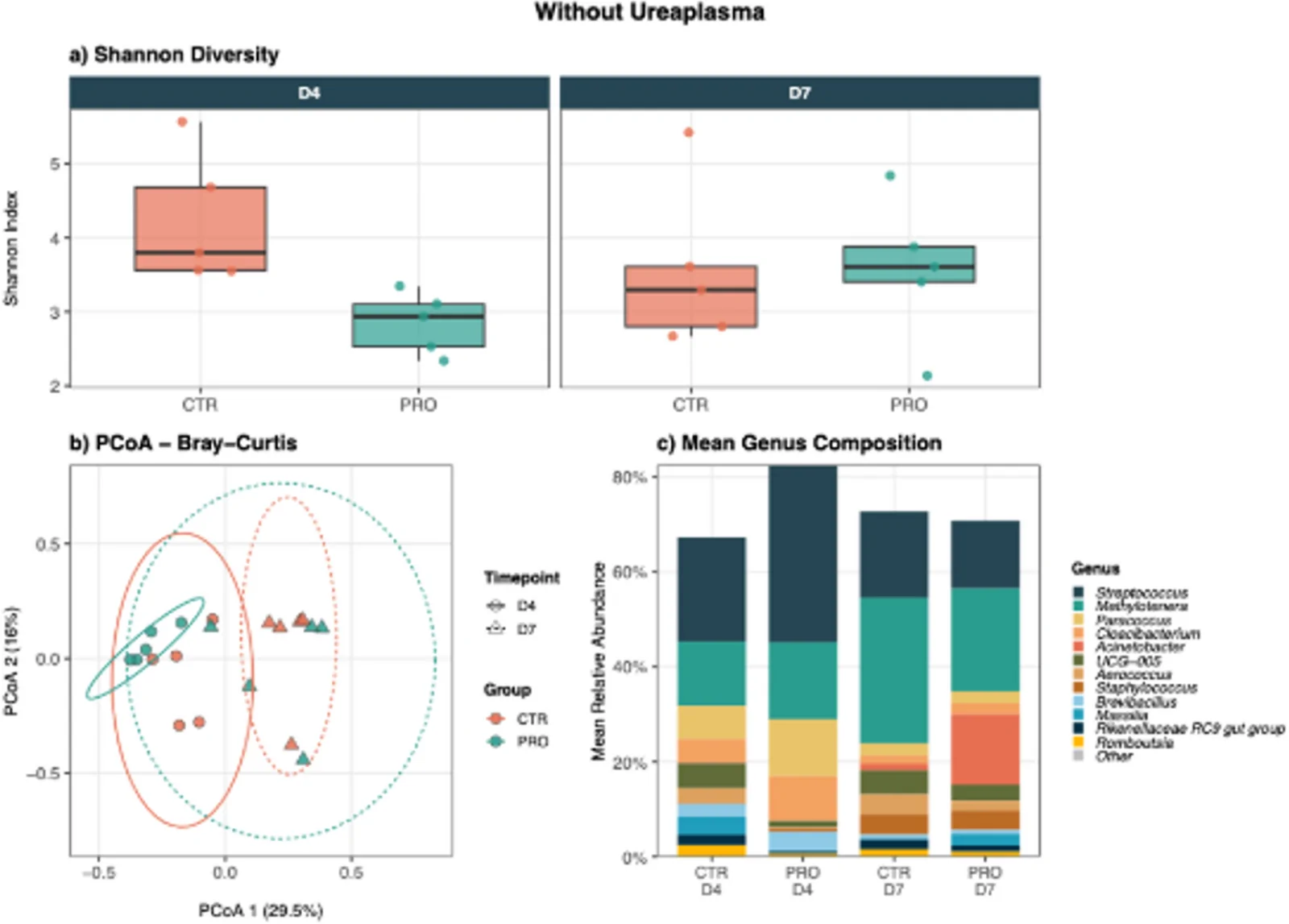
Shannon Diversity (**a**), PCoA – Bray- Curtis (**b**) and Mean Genus Composition (**c**) without *Ureaplasma* from samples collected on Day 4 and Day 7 after treatment administration in the Control (CTR; *n* = 5) and Probiotic (PRO; *n* = 5) groups

The PRO group showed lower alpha diversity compared to the CTR group across all evaluated metrics (Online Resource 3). Unpaired Wilcoxon tests indicated nominally significant differences at D4 for Shannon (W = 1, *p* = 0.022; Fig. 4A), Observed ASVs (W = 2, *p* = 0.037), Chao1 (W = 2, *p* = 0.037), and Simpson (W = 2, *p* = 0.037). However, after adjustment for multiple testing using the Benjamini–Hochberg procedure, none of the comparisons remained statistically significant (adjusted *p* = 0.073 for all metrics). In D7, no differences between groups were observed for any alpha diversity metric (*p* > 0.40) (Online Resource 4). In the analysis excluding *Ureaplasma*, the signal at D4 was slightly strengthened for the Shannon index (W = 0, *p* = 0.012; Fig. 5A), although it remained non-significant after multiple testing correction.

The paired analysis comparing the temporal change (ΔD7–D4) between groups detected significant differences for Shannon (W = 1, *p* = 0.02), Observed ASVs (W = 2, *p* = 0.04), Chao1 (W = 2, *p* = 0.04), and Simpson (W = 2, *p* = 0.04) at D4 (Online Resource 5). However, after adjustment for multiple testing using the Benjamini–Hochberg procedure, none of the comparisons remained statistically significant (adjusted *p* = 0.07 for all metrics). In D7, no differences between groups were observed for any alpha diversity metric (*p* > 0.40). Likewise, the paired comparison between D4 and D7 within each group revealed no significant changes in alpha diversity over time in either group (adjusted *p* > 0.44; Online Resource 6).

#### Beta-diversity

Principal Coordinates Analysis (PCoA) based on Bray–Curtis dissimilarity did not reveal clear separation between PRO and CTR groups at either timepoint (Fig. 4B; Online Resource 7). However, PERMANOVA with permutations constrained by animal (paired design) detected a significant effect of the group × time point model on microbial community composition using the Bray–Curtis distance metric (R² = 0.197, F = 1.31, *p* = 0.004; Online Resource 8). This finding was consistent in the dataset excluding *Ureaplasma* (Fig. 5B), where the effect was even more pronounced (R² = 0.318, F = 2.49, *p* = 0.004), indicating that removal of the highly variable dominant taxon increased the ability to detect differences in the underlying microbial community.

Notably, PERMANOVA performed without constraining permutations by animal did not reach statistical significance (*p* = 0.183), demonstrating that the repeated-measures design was essential for detecting the effect of probiotic treatment by accounting for inter-individual variability. In contrast, PERMANOVA based on the Aitchison distance (CLR-transformed data with Euclidean distance) using constrained permutations was not significant (*p* = 0.185), suggesting that the Bray–Curtis metric was more sensitive for detecting compositional differences in this dataset.

#### Taxonomic composition

The vaginal microbiota was dominated at the genus level by *Ureaplasma* (mean relative abundance: 29.9% in the PRO group vs. 9.5% in the CTR group at D4), *Streptococcus* (25.2% vs. 19.4% at D4), *Methylotenera* (11.4% vs. 12.6% at D4), *Paracoccus* (8.3% vs. 6.3% at D4), and *Cloacibacterium* (6.5% vs. 4.3% at D4) (Fig. 4C; Online Resource 9–10).

Notably, *Ureaplasma* exhibited high inter-individual variability within the PRO group (SD = 38.9%), suggesting that this taxon may dominate the community in a sample-specific manner. This marked between-animal variation is consistent with previous reports in cattle, in which *Ureaplasma* is repeatedly identified as a dominant vaginal genus with abundances that differ widely between individual cows (Bicalho et al. 2017); group-level means for this taxon should therefore be interpreted with caution, which is why all analyses were also repeated after excluding *Ureaplasma*. The presence of *Methylotenera* and *Paracoccus*, genera typically associated with environmental sources, may be related to water or environmental contamination during sampling, although they have also been reported in bovine mucosal microbiomes. Because these genera are also well-documented reagent and laboratory contaminants in low-biomass amplicon sequencing (Salter et al. 2014), their presence should be interpreted cautiously and, where possible, cross-checked against negative extraction and PCR controls before being attributed to the vaginal community itself.

#### Differential abundance

ANCOM-BC2 analysis did not identify any genera with significantly different abundance between PRO and CTR groups at either timepoint (q > 0.05 for all taxa; Online Resource 11). At D4, the genera with the lowest q-values were *Clostridium* sensu stricto 1 (LFC = − 0.68, q = 0.223), NK4A214 group (LFC = − 0.60, q = 0.223).

Lower q-values were observed (*Clostridium* sensu stricto 1: q = 0.136; NK4A214 group: q = 0.136) in the dataset excluding *Ureaplasma*, suggesting that removal of the dominant taxon improved the resolution of the analysis, although statistical significance was still not observed (Online Resource 12).

No genus reached statistical significance after Benjamini–Hochberg correction in ALDEx2 analysis. However, effect size analysis identified biologically relevant candidate taxa at D4. *Lacticacilseibalus*, a probiotic genus, was enriched in the PRO group and exhibited the largest effect size among all genera (effect size = 2.01, uncorrected *p* = 0.014), representing the greatest difference between groups. In contrast, *Bradyrhizobium* (effect size = − 1.22) and the UCG-009 group (effect size = − 1.32) were enriched in the CTR group. At D7, no taxon exhibited a notable effect size (Online Resource 13).

## Discussion

In this study, using a progesterone-exposed cow model, we tested the effect of intravaginal probiotics on the modulation of the local microbiota. The main findings were as follows: (1) the isolation of *Lactobacillus rhamnosus* up to 4 days post treatment, together with the enrichment of *Lacticaseibacillus* at D4 in the PRO group, suggests that this strain could have remained viable in the vaginal; (2) there were no changes in vaginal pH between groups; and (3) no significant differential abundance of any genera was observed between PRO and CTR groups at either timepoint; however, a significant effect of the group and timepoint on microbial community composition was detected, enhanced while excluding *Ureaplasma* from the dataset.

To better understand the persistence of the administered probiotic strains, we relied on microbiological culture techniques. Several researchers have pointed out that only 1% to 2% of microorganisms can be detected by traditional culture media (Torsvik et al. 2002). In this study, we used microbiological culture methods to estimate the persistence of the LAB used in the reproductive tract of cows. Our culture results indicate that they could be remained in high abundance in the vaginal environment until D4. After this time point, they were no longer detected by traditional in vitro culture methods. We hypothesize that after this period, there was cell death and/or competition exclusion by autochthonous microorganisms of the bovine vaginal microbiota. These findings are relevant for future studies and suggest the need for repeated administration of probiotic capsules. This has already been reported by other researchers, who suggest weekly applications (Ametaj et al. 2014; Deng et al. 2015, 2016). However, in the identification of the species by MALDI-TOF, the *L. reuteri* strain was not detected. We propose that there was reduced viability and/or competition interactions with the *L. rhamnosus* strain, although these strains have previously been evaluated in combination (Drago et al. 2020).

To select the LAB strains used in this study, we considered species previously isolated from the vaginal mucosa of cows and women, such as: *L. rhamnosus*,* L. reuteri*,* L. gasseri*,* L. acidophillus* (Wang et al. 2013; Swartz et al. 2014; Graham et al. 2021; Shen et al. 2022). In addition, we selected commercially available strains with proven probiotic action. Thus, we used a formulation of lyophilized strains of *L. rhamnosus* (LR04, LR06) and *L. reuteri* (LRE 02). These strains have been validated in inhibition assays against *Escherichia coli* (Mogna et al. 2012; Chamignon et al. 2020) and have previously been used in combination (Drago et al. 2020). Genís et al. (2018) showed that the combination of probiotic strains of *L. rhamnosus*,* Pediococcus acidilactici* and *L. reuteri* administered intravaginally reduced the incidence of uterine infections in dairy cows by 58%. Another study using similar probiotic strains reported a decrease in the incidence of metritis and endometritis in one of the farms studied (Madureira et al. 2023). For future studies, we suggest that cows be experimentally challenged with pathogenic *Escherichia coli* strains, since in the present study no *E. coli* was detected in the vaginal microbiota of the sampled cows. This should be interpreted as the absence of ASVs classified to the Escherichia/Shigella genus rather than as definitive species-level identification, since short V3-V4 amplicons classified with a naïve Bayesian classifier typically do not resolve 16 S rRNA gene sequences to the species level, particularly within taxonomically challenging groups such as the *Enterobacteriaceae* (Johnson et al. 2019).

Despite the promising background of these LAB strains and their potential to influence the vaginal environment, no significant changes in vaginal pH were observed in our experiment. It was expected that the PRO group would exhibit a lower vaginal pH than the CTR group, considering the previously reported effects of these microorganisms on vaginal pH. However, no significant differences were observed in mean vaginal pH between the CTR and PRO groups (*P* > 0.05; 6.8 ± 0.2 and 6.9 ± 0.1, respectively). Unlike women, the vaginal pH of cows varies from neutral to slightly alkaline, ranging from 6.7 to 7.5 (Beckwith-Cohen et al. 2012; O’Hanlon et al. 2013; Swartz et al. 2014). In this context, it is important to note that while *Lactobacillus* spp. are aciduric, their metabolic optimum is often closer to neutral pH. For instance, *L. rhamnosus* has shown optimal growth kinetics at pH 6.9 (Liew et al. 2005), and the genus generally exhibits optimal growth between pH 5.5 and 6.2 (Hammes and Hertel 2015). Therefore, the neutral pH observed in our study suggests that the bovine vaginal environment supports the viability of these strains without necessarily inducing the marked acidification typical of the human vagina. Instead, these bacteria likely face a highly competitive environment where nutrient availability, rather than acidity, may represent the primary limiting factor. Additionally, regarding data interpretation, an important point in the site of pH measurement. Previous studies have evaluated different anatomical regions, such as the vestibule (caudal vagina), mid-vaginal region, cranial vagina and cervical fornix, reporting differences in pH values between the sites (Beckwith-Cohen et al. 2012), as well as variation depending on the methods used for pH assessment.

Hormonal status is another important physiological factor that influences the vaginal environment. The taxonomic composition of the vaginal microbiota changes according to the phase of the estrous cycle (De Ruiz Holgado and Nader-Macias 1999; Otero et al. 2000; Reid 2001). In this study, we chose to synchronize the animals using long-acting injectable progesterone to avoid the influence of the estrous cycle on the vaginal microbiota, which may have indirectly influenced the survival and establishment of the administered probiotics. However, it is important to acknowledge that, while the use of non-pregnant synchronized cows provides a controlled model, it does not fully represent the postpartum condition. Unlike the animals in this study, postpartum cows experience immunosuppression and active uterine involution, physiological conditions that could alter host-probiotic interaction.

In cattle, the genus *Lactobacillus* has consistently been reported at low relative abundance during the estrous cycle, being more abundant in the follicular phase than in the luteal phase (Quereda et al. 2020). *Lactobacillus* species are common in the vaginal microbiota of cows, being detected in 80% of vaginal samples from beef cows. However, they were found at a low relative abundance (0.36 ± 0.66) of the total population of the 16 S rRNA gene (Swartz et al. 2014). In another study in dairy heifers, the genus *Lactobacillus* was detected in 37% (7/19) and 10% (2/20) of the samples collected in the follicular and luteal periods, respectively, and was found at low relative abundance (0.19 ± 0.04%). In this study, among all collection days, the genus *Lactobacillus* was most frequently isolated on D4 (81.8%; 9/11) after treatment. These findings suggest a treatment-induced effect, as reflected by the changes in microbial population dynamics observed on D4 following the intervention in PRO but not in the CTR group. In addition, differential abundance analysis revealed an enrichment of *Lacticaseibacillus* in the PRO group, providing further evidence that these changes were associated with the LAB treatment. It is worth clarifying that the apparent difference in genus names between the culture-based/MALDI-TOF identification (*Lactobacillus rhamnosus*) and the amplicon-based differential abundance result (*Lacticaseibacillus*) is nomenclatural rather than biological. The 2020 reclassification of the genus *Lactobacillus* transferred *L. rhamnosus*, together with the other former members of the *L. casei* group, into the novel genus *Lacticaseibacillus* (Zheng et al. 2020), which is the nomenclature adopted by the SILVA v138.1 reference database used for taxonomic assignment in this study, while the commercial probiotic and the MALDI-TOF reference library still use the legacy name. The culture-based and amplicon-based results are therefore consistent and mutually reinforcing rather than discordant, and we recommend that future studies of this kind explicitly harmonize genus-level nomenclature between culture-dependent and molecular datasets to avoid this potential source of confusion for readers.

The microbiome of healthy cows is typically enriched with bacteria from the phylum *Firmicutes*, whereas cows that develop metritis or endometritis tend to show a higher relative abundance of *Bacteroidota* (Miranda-CasoLuengo et al. 2019; Galvão et al. 2019; Adnane and Chapwanya 2022; Tasara et al. 2023). The taxa identified at higher relative abundance in the samples were *Ureaplasma*,* Streptococcus*,* Methylotenera*,* Paracoccus*,* and Cloacibacterium*. *Ureaplasma* is the most commonly found genus, regardless of the health and reproductive status of the animals (Bicalho et al. 2017; Galvão et al. 2019). According to Kudo et al. (2021), the genus *Ureaplasma* was predominant in the vaginal microbiota of healthy cows and was negatively correlated with other bacteria. *Ureaplasma diversum* has been previously reported in the vaginal microbiota of cows in several countries, including Brazil (Azevedo et al. 2017; Nascimento-Rocha et al. 2017). Bacteria of the genus *Streptococcus* are considered part of the resident microbiota of the vulva, vagina, skin, and gastrointestinal tract (Oliveira 2012). Several authors have reported the isolation of *Streptococcus* spp. as commensal and opportunistic microorganisms, often detected in the presence of pathogenic bacteria without causing harm to the animal’s health (Panangala et al. 1978; Kudo et al. 2021; Adnane and Chapwanya 2022). Similar results were observed using standard culture medium, in which *Streptococcus* was identified in vaginal swab samples. Some studies have associated *α-hemolytic Streptococcus* with probiotic characteristics in the vaginal microbiota, suggesting a potential role in preventing reproductive tract infections, such as metritis (Otero et al. 2000). However, other authors have reported divergent findings, suggesting that this microorganism may favor this proliferation of more pathogenic bacteria due to the production of penicillinase, which can protect pathogens associated with uterine diseases (Silva and Lobato 1999; Sheldon et al. 2002). In this study, *Streptococcus* was interpreted as an opportunistic commensal component of vaginal microbiota, since all animals included in the experiment were clinically healthy.

Following the characterization of microbial taxa, the analysis of alpha and beta diversity revealed important insights into the short-term effects of intravaginal probiotic administration on the vaginal microbiota of dairy cows. On D4, the CTR group exhibited significantly greater richness and diversity, as evidenced by higher values in most alpha diversity indices, including observed ASVs, Fisher’s alpha, Simpson’s index, Shannon index, and Pielou’s evenness. This indicates that the probiotic treatment may have temporarily reduced microbial diversity, potentially due to transient dominance or competitive interactions involving the administered strains. However, these differences were no longer evident by D7, suggesting a re-equilibration of the microbial community over time. In contrast to Toquet et al. (2025), who reported that two administrations of a *Lactobacillus*-based probiotic before artificial insemination increased both alpha and beta diversity at pregnancy diagnosis in one sheep herd, this effect was not observed consistently across all herds. Interestingly, despite these transient effects on alpha diversity, no significant differences were observed in beta diversity at either time point. This indicates that the overall microbial composition between groups remained relatively stable and was not substantially altered by the probiotic treatment. The overlapping ellipses in the beta diversity ordinary plots (e.g., PCoA) further support this conclusion, suggesting that while local diversity metrics may fluctuate, the global structure of the vaginal microbiota was resilient to short-term probiotic intervention under the conditions tested. A study conducted by Pascottini et al. (2020) demonstrated that days in milk (DIM; 10, 21, and 35) had no significant effect on either alpha or beta diversity of the uterine microbiota. However, clear distinctions were observed based on uterine health status. Cows with clinical endometritis exhibited a distinct microbial profile compared to healthy cows and those with subclinical endometritis. Healthy cows and those with subclinical endometritis showed greater alpha and beta diversity than cows with clinical endometritis, suggesting that reduced microbial diversity may be associated with the development of clinical endometritis.

The absence of statistically significant differences in microbial composition at either Day 4 or Day 7 days suggests that the formulation used did not induce measurable modulation of the resident community within this timeframe. Also, the sample size might have reduced the power to detect differences at the individual taxon level, even though global compositional differences were detectable by paired PERMANOVA. The enrichment of *Lacticaseibacillus* in the PRO group is biologically consistent with the probiotic intervention and warrants further investigation in studies with greater statistical power. Microbial shifts may occur outside of the evaluated window, either at earlier or later time points, or a longer treatment duration may be required to promote stable colonization and impact. In addition, the LAB strain combination, concentration, and formulation may not have been optimal for establishing colonization in the vaginal environment of non-lactating, hormonally synchronized cows. Modifying the dose, adjusting the formulation, altering the strains used, or applying the treatment to cows in different physiological stages, such as those in the transition period, may increase the likelihood of detecting probiotic induced changes in microbial profiles. Despite the absence of microbiota shifts, a significant increase in bacilli growth observed through culture on D4 suggests that transient colonization may have occurred, although this colonization was not sustained or sufficient to alter the overall bacterial community composition. These transient interactions may still have functional relevance, for example, through modulation of mucosal immunity or competitive exclusion of pathogens. To the best of our knowledge, this is the first study to combine targeted culture-based recovery of lactic acid bacteria with 16 S rRNA gene sequencing to monitor the vaginal environment of cows treated with probiotic strains, and it opens new avenues for future research in this field. To date, the reduction in the incidence of postpartum diseases of dairy cows observed by other authors (Ametaj et al. 2014; Deng et al. 2015; Genís et al. 2017) after the use of probiotics cannot be fully explained by detectable changes in the microbiome. Two intrinsic limitations of the amplicon approach also bear on the interpretation of the absence of modulation. First, 16 S rRNA gene surveys yield relative rather than absolute abundances, so a real increase in lactobacilli biomass, such as that indicated here by culture, may go undetected if total bacterial load changes concurrently. Second, variation in 16 S rRNA gene copy number between taxa distorts abundance estimates and still lacks a reliable correction (Louca et al. 2018). Complementing amplicon data with quantitative approaches, such as qPCR of total bacterial load or of strain-specific targets, would let future studies distinguish an absence of colonization from an absence of detectable compositional change. Local effects on the vaginal microenvironment may contribute to the modulation of inflammatory responses. Relying solely on antimicrobial drugs is insufficient to curb the rise of antimicrobial resistance; therefore, the development of alternative therapeutic strategies for diseases in farm animals is essential to safeguard both animal and human health.

Treatment with probiotic LAB strains did not modulate the vaginal microbiota at Day 4 and Day 7, nor did it alter vaginal pH up to Day 14. *Lactobacillus rhamnosus* administered in the PRO group remained viable until Day 4, whereas *Lactobacillus reuteri* was not detected in the vaginal mucosa of the cows after treatment. To the best of our knowledge, this is the first study to characterize the vaginal microbiota of cows treated with *Lactobacillus* probiotic strains using both culture-dependent and molecular approaches, providing novel insights for future investigations on the influence of probiotics on bovine reproductive health.

## Supplementary Information

Below is the link to the electronic supplementary material.

Supplementary Material 1

Supplementary Material 2

Supplementary Material 3

Supplementary Material 4

Supplementary Material 5

Supplementary Material 6

Supplementary Material 7

Supplementary Material 8

Supplementary Material 9

Supplementary Material 10

Supplementary Material 11

Supplementary Material 12

Supplementary Material 13

## Data Availability

The data that support the findings of this study are available from the corresponding author upon reasonable request.
