# Supplementary material for: Characterization of the vaginal microbiota of dairy cows treated with probiotics
Source: Vet Res Commun. 2026 Nov 11;51(1):48. doi: 10.1007/s11259-026-11616-3 (PMC13751720; doi:10.1007/s11259-026-11616-3)
Supplement: Supplementary file 5 [file 11259_2026_11616_MOESM1_ESM.pdf]

Veterinary Research Communications

Characterization of the vaginal microbiota of dairy cows treated with probiotics

Aline Policarpo Baioco[1], Fabiane Pereira de Moraes[2], Sergio Farias Vargas Júnior[2], Arnaldo Diniz Vieira[2], Rafael Gianella Mondadori[2], Fábio Pereira Leivas Leite[3], Jeferson Vidart Ramos[4], José Mário Barichello[5], Ligia Margareth Cantarelli Pegoraro[6], Bernardo Garziera Gasperin[2], André Gustavo Cabrera Dalto[1], Monique Tomazele Rovani[1*]

[1]Programa de Pós-graduação em Ciências Veterinária (PPGCV), Universidade Federal do Rio Grande do Sul (UFRGS), Porto Alegre, RS, Brasil; [2]Programa de Pós-graduação em Veterinária (PPGV), Universidade Federal de Pelotas (UFPel), Pelotas, RS, Brasil; [3]Programa de Pós-graduação em Biotecnologia (PPGBiotec), Universidade Federal de Pelotas (UFPel), Pelotas, RS, Brasil; [4]Programa de Pós-Graduação em Microbiologia e Parasitologia (PPGMPar), Universidade Federal de Pelotas (UFPel), Pelotas, RS, Brasil; [5]Centro de Ciências Químicas, Farmacêuticas e de Alimentos (CCQFA), Universidade Federal de Pelotas, Pelotas, RS, Brasil; [6]Embrapa Clima Temperado, Pelotas, RS, Brasil.
*Corresponding Author: Monique Tomazele Rovani, monique.rovani@ufrgs.br

Supplementary Information

Online Resource 1 Tracking of sequencing reads across the DADA2 pipeline steps from samples collected on Day 4 and Day 7 after treatment administration in the Control (CTR; n = 5) and Probiotic (PRO; n = 5) groups

SAMPLE	GROUP	INPUT	FILTERED	MERGED	W/O QUIMERAS
11887_D4	PRO	277.955	210.940	199.885	178.537
11888_D4	PRO	187.080	140.052	132.673	121.498
11889_D4	PRO	278.203	210.784	200.171	182.237
11890_D4	PRO	371.431	283.910	275.740	252.067
11891_D4	PRO	326.286	260.928	256.380	251.486
11892_D4	CTR	167.667	125.222	117.731	108.641
11893_D4	CTR	293.303	221.286	208.550	189.153
11894_D4	CTR	381.402	295.280	250.436	228.123
11895_D4	CTR	225.341	170.254	157.168	134.947
11896_D4	CTR	374.632	286.435	255.469	220.608
11877_D7	PRO	25.519	22.530	20.743	20.145
11878_D7	PRO	44.345	39.037	34.286	33.110
11879_D7	PRO	180.618	158.516	152.891	141.792
11880_D7	PRO	107.979	95.217	87.769	80.723
11881_D7	PRO	66.044	58.158	57.322	56.984
11882_D7	CTR	78.819	69.454	56.816	54.145
11883_D7	CTR	22.079	19.249	17.255	16.072
11884_D7	CTR	68.405	60.211	59.439	59.015
11885_D7	CTR	34.043	30.022	28.642	26.340
11886_D7	CTR	13.564	11.893	10.764	10.216
