# Supplementary material for: Characterization of the vaginal microbiota of dairy cows treated with probiotics
Source: Vet Res Commun. 2026 Nov 11;51(1):48. doi: 10.1007/s11259-026-11616-3 (PMC13751720; doi:10.1007/s11259-026-11616-3)
Supplement: Supplementary file 8 [file 11259_2026_11616_MOESM4_ESM.pdf]

Veterinary Research Communications

Characterization of the vaginal microbiota of dairy cows treated with probiotics

Aline Policarpo Baioco[1], Fabiane Pereira de Moraes[2], Sergio Farias Vargas Júnior[2], Arnaldo Diniz Vieira[2], Rafael Gianella Mondadori[2], Fábio Pereira Leivas Leite[3], Jeferson Vidart Ramos[4], José Mário Barichello[5], Ligia Margareth Cantarelli Pegoraro[6], Bernardo Garziera Gasperin[2], André Gustavo Cabrera Dalto[1], Monique Tomazele Rovani[1*]

[1]Programa de Pós-graduação em Ciências Veterinária (PPGCV), Universidade Federal do Rio Grande do Sul (UFRGS), Porto Alegre, RS, Brasil; [2]Programa de Pós-graduação em Veterinária (PPGV), Universidade Federal de Pelotas (UFPel), Pelotas, RS, Brasil; [3]Programa de Pós-graduação em Biotecnologia (PPGBiotec), Universidade Federal de Pelotas (UFPel), Pelotas, RS, Brasil; [4]Programa de Pós-Graduação em Microbiologia e Parasitologia (PPGMPar), Universidade Federal de Pelotas (UFPel), Pelotas, RS, Brasil; [5]Centro de Ciências Químicas, Farmacêuticas e de Alimentos (CCQFA), Universidade Federal de Pelotas, Pelotas, RS, Brasil; [6]Embrapa Clima Temperado, Pelotas, RS, Brasil.
*Corresponding Author: Monique Tomazele Rovani, monique.rovani@ufrgs.br

Online Resource 4 Unpaired Wilcoxon tests for Alpha-Diversity with full dataset from samples collected on Day 4 and Day 7 after treatment administration in the Control (CTR; n = 5) and Probiotic (PRO; n = 5) groups

Timepoint	Metrics	W	p-value	adjusted p
D4	Observed	2	0,037	0,073
D4	Chao1	2	0,037	0,073
D4	Shannon	1	0,022	0,073
D4	Simpson	2	0,037	0,073
D7	Observed	17	0,403	0,531
D7	Chao1	17	0,403	0,531
D7	Shannon	16	0,531	0,531
D7	Simpson	16	0,531	0,531
