# Supplementary material for: Characterization of the vaginal microbiota of dairy cows treated with probiotics
Source: Vet Res Commun. 2026 Nov 11;51(1):48. doi: 10.1007/s11259-026-11616-3 (PMC13751720; doi:10.1007/s11259-026-11616-3)
Supplement: Supplementary file 9 [file 11259_2026_11616_MOESM5_ESM.pdf]

Veterinary Research Communications

Characterization of the vaginal microbiota of dairy cows treated with probiotics

Aline Policarpo Baioco[1], Fabiane Pereira de Moraes[2], Sergio Farias Vargas Júnior[2], Arnaldo Diniz Vieira[2], Rafael Gianella Mondadori[2], Fábio Pereira Leivas Leite[3], Jeferson Vidart Ramos[4], José Mário Barichello[5], Ligia Margareth Cantarelli Pegoraro[6], Bernardo Garziera Gasperin[2], André Gustavo Cabrera Dalto[1], Monique Tomazele Rovani[1*]

[1]Programa de Pós-graduação em Ciências Veterinária (PPGCV), Universidade Federal do Rio Grande do Sul (UFRGS), Porto Alegre, RS, Brasil; [2]Programa de Pós-graduação em Veterinária (PPGV), Universidade Federal de Pelotas (UFPel), Pelotas, RS, Brasil; [3]Programa de Pós-graduação em Biotecnologia (PPGBiotec), Universidade Federal de Pelotas (UFPel), Pelotas, RS, Brasil; [4]Programa de Pós-Graduação em Microbiologia e Parasitologia (PPGMPar), Universidade Federal de Pelotas (UFPel), Pelotas, RS, Brasil; [5]Centro de Ciências Químicas, Farmacêuticas e de Alimentos (CCQFA), Universidade Federal de Pelotas, Pelotas, RS, Brasil; [6]Embrapa Clima Temperado, Pelotas, RS, Brasil.
*Corresponding Author: Monique Tomazele Rovani, monique.rovani@ufrgs.br

Online Resource 5 Paired analysis of Alpha-Diversity with full dataset comparing the temporal change (ΔD7–D4) between groups from samples collected on Day 4 and Day 7 after treatment administration in the Control (CTR; n = 5) and Probiotic (PRO; n = 5) groups

Timepoint	Metric	p-value	W	adjusted p
**D4**	Observed	0.04	2	0.07
**D4**	Chao1	0.04	2	0.07
**D4**	Shannon	0.02	1	0.07
**D4**	Simpson	0.04	2	0.07
**D7**	Observed	0.4	17	0.53
**D7**	Chao1	0.4	17	0.53
**D7**	Shannon	0.53	16	0.53
**D7**	Simpson	0.53	16	0.53
