# Supplementary material for: Characterization of the vaginal microbiota of dairy cows treated with probiotics
Source: Vet Res Commun. 2026 Nov 11;51(1):48. doi: 10.1007/s11259-026-11616-3 (PMC13751720; doi:10.1007/s11259-026-11616-3)
Supplement: Supplementary file 13 [file 11259_2026_11616_MOESM9_ESM.pdf]

Veterinary Research Communications

Characterization of the vaginal microbiota of dairy cows treated with probiotics

Aline Policarpo Baioco[1], Fabiane Pereira de Moraes[2], Sergio Farias Vargas Júnior[2], Arnaldo Diniz Vieira[2], Rafael Gianella Mondadori[2], Fábio Pereira Leivas Leite[3], Jeferson Vidart Ramos[4], José Mário Barichello[5], Ligia Margareth Cantarelli Pegoraro[6], Bernardo Garziera Gasperin[2], André Gustavo Cabrera Dalto[1], Monique Tomazele Rovani[1]*

[1]Programa de Pós-graduação em Ciências Veterinária (PPGCV), Universidade Federal do Rio Grande do Sul (UFRGS), Porto Alegre, RS, Brasil; [2]Programa de Pós-graduação em Veterinária (PPGV), Universidade Federal de Pelotas (UFPel), Pelotas, RS, Brasil; [3]Programa de Pós-graduação em Biotecnologia (PPGBiotec), Universidade Federal de Pelotas (UFPel), Pelotas, RS, Brasil; [4]Programa de Pós-Graduação em Microbiologia e Parasitologia (PPGMPar), Universidade Federal de Pelotas (UFPel), Pelotas, RS, Brasil; [5]Centro de Ciências Químicas, Farmacêuticas e de Alimentos (CCQFA), Universidade Federal de Pelotas, Pelotas, RS, Brasil; [6]Embrapa Clima Temperado, Pelotas, RS, Brasil.
*Corresponding Author: Monique Tomazele Rovani, monique.rovani@ufrgs.br

Online Resource 9 Relative abundance of phylum (a), and genus (b) in each sample collected on Day 4 and Day 7 after treatment administration in the Control (CTR; n = 5) and Probiotic (PRO; n = 5) groups

a

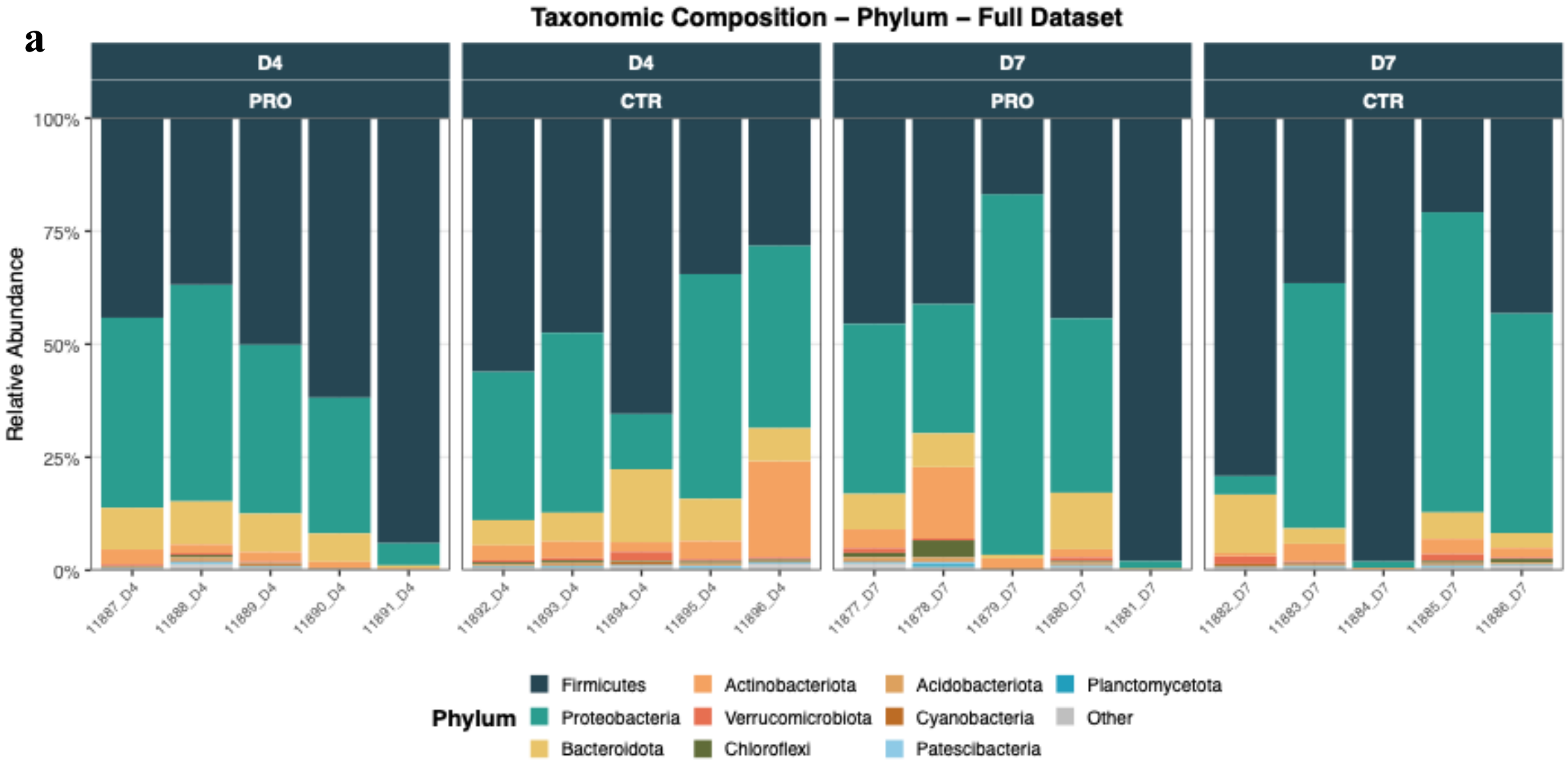

b

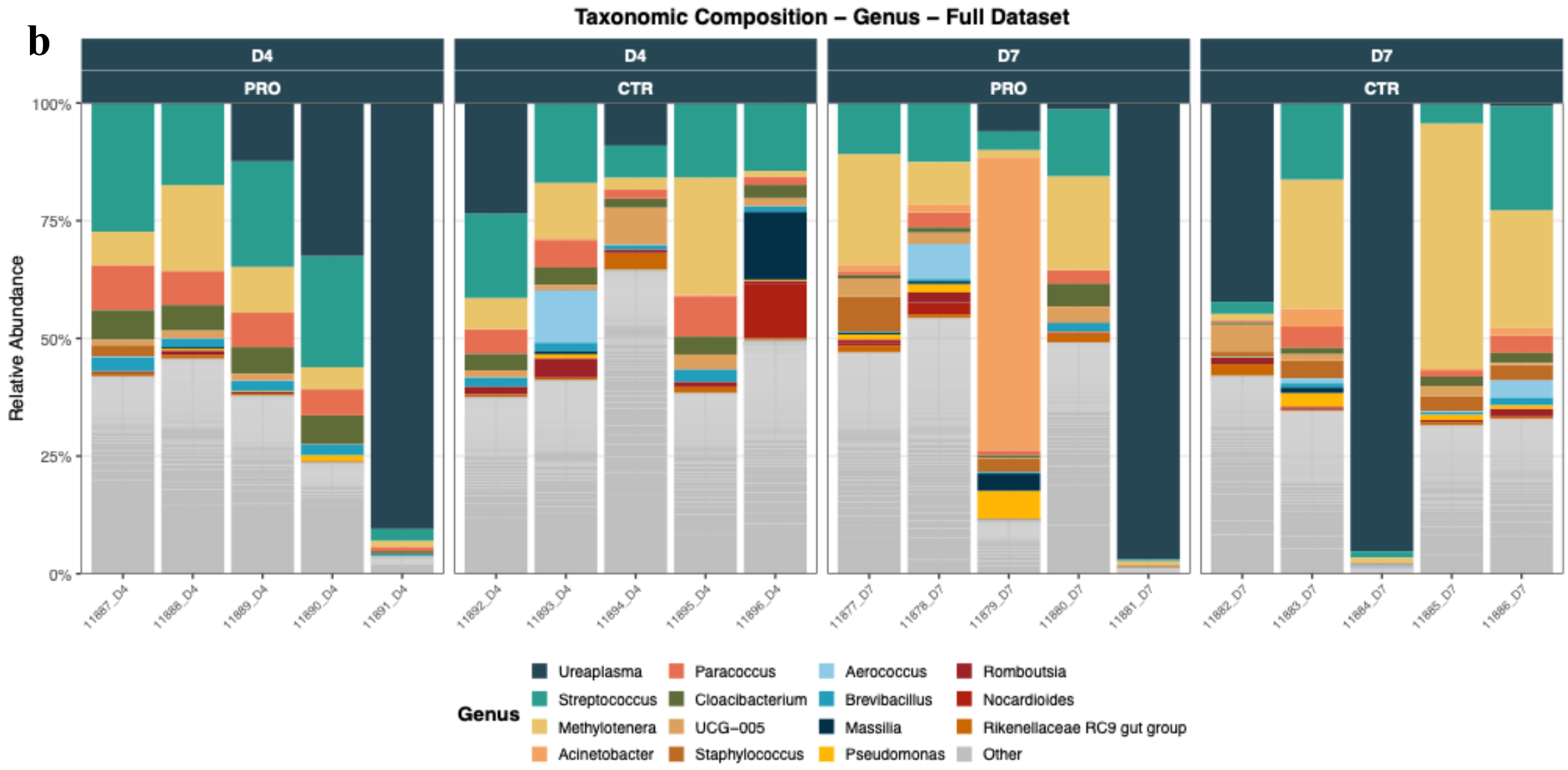

Taxonomic Composition – Genus – Full Dataset
D4
PRO
D4
CTR
D7
PRO
D7
CTR
Relative Abundance
100%
75%
50%
25%
0%
11887_D4
11888_D4
11889_D4
11890_D4
11891_D4
11892_D4
11893_D4
11894_D4
11895_D4
11896_D4
11877_D7
11878_D7
11879_D7
11880_D7
11881_D7
11882_D7
11883_D7
11884_D7
11885_D7
11886_D7
Genus
Ureaplasma
Streptococcus
Methylotenera
Acinetobacter
Paracoccus
Cloacibacterium
UCG−005
Staphylococcus
Aerococcus
Brevibacillus
Massilia
Pseudomonas
Romboutsia
Nocardioides
Rikenellaceae RC9 gut group
Other
